# Addressing Power of Equivalence Tests for Statistical Fairness Assessments of Machine Learning Models in Healthcare

**DOI:** 10.3390/cancers18213514

**Published:** 2026-10-31

**Authors:** David Watson, Momin M. Malik, Jennifer Le-Rademacher, Mahmoud AlJuhani, Shauna M. Overgaard, Chung-Il Wi, Young J. Juhn

**Affiliations:** 1 Department of Quantitative Health Sciences, Mayo Clinic, Rochester, MN 55905, USA; 2 Precision Population Science Lab, Mayo Clinic, Rochester, MN 55905, USA; 3 Department of Pediatric and Adolescent Medicine, Mayo Clinic, Rochester, MN 55905, USA; 4 Center for Digital Health, Mayo Clinic, Rochester, MN 55905, USA

**Keywords:** artificial intelligence, hypothesis test, fairness audit, oncology

## Abstract

**Background/Objectives**: Machine learning and artificial intelligence (AI) have substantial potential to change the practice of oncology through individualized patient care; however, it is important to ensure that any benefits are realized equitably. There are several examples of AI tools exhibiting bias towards traditionally under-resourced or marginalized groups. Proper validation that these models perform well across populations requires rigorous statistical design and interpretation. One component that is currently lacking in fairness assessments is a clear, prespecified definition of the evidence required to conclude that an AI tool is fair. **Methods**: To address this issue, we embed the notion of fairness assessment of an AI model within the *statistical equivalence testing* framework. This approach provides an a priori definition of fairness and an explicit decision rule for concluding, with a high degree of confidence, that model performance falls within a prespecified margin. The framework also supplies power and sample size calculations to support the design phase of a fairness assessment, which we develop for the case of modeling a binary outcome and a binary classification. As a demonstration, we use a “four-fifths” rule of thumb to set equivalence margins but emphasize the necessity of clinical and ethical context for choosing the margins in practice. **Results**: Using two published oncology models, we show that a realistic sample size can be adequate for some performance metrics and inadequate for others. We also address power and sample size considerations for asymmetric association measures and impossibility results for fairness. **Conclusions**: Assessments of AI performance fairness should be designed with intention such that target metrics and equivalence margins are specified in advance with ample sample size to conclude fairness.

## Simple Summary

Artificial intelligence (AI) has the potential to improve cancer detection, risk prediction, and personalized care, but it is important to ensure that AI models perform fairly across different patient populations. Current fairness assessments often report differences between groups or conclude that models are fair only based on effect size, or when the difference is not statistically significant. However, a lack of significant difference does not necessarily prove comparable performance. This manuscript proposes using *equivalence testing*, which requires researchers to define in advance how much difference between groups would still be considered acceptable. The approach also incorporates sample size and power calculations to determine whether available data is sufficient to evaluate fairness. Examples from oncology models show that researchers may have enough data to establish fairness for some performance measures but not others, emphasizing the importance of defining fairness criteria and evidence requirements before conducting an assessment.

## 1. Introduction

Artificial intelligence (AI) is changing the practice of oncology with substantial potential to provide individualized care to patients through earlier cancer detection and risk assessment [1,2]. A major challenge is ensuring that any benefits are equitably realized across the entire population [3,4]. There are several examples of AI tools and prediction models exhibiting bias against traditionally under-resourced or marginalized groups both within oncology and in medicine more generally. Given the different uses of the term bias [5], here we specify that we mean *differential model performance* that could further disadvantage patients who occupy one or more marginalized social positions. Examples of differential performance include differential racial impact of cancer screening eligibility and model-based prediction of lung cancer risk [6,7,8], differential performance of dermatology AI models by skin tone [9,10], and misdiagnosis of computational pathology models for carcinomas by demographics and race [11,12,13]. Reasons for such disparities range from the effects of structural racism embedded in proxy outcome variables [14,15], to data missingness [16], and to class imbalance among underrepresented groups [17].

A key step for ensuring equitable AI is proper validation that these models perform well across a range of diverse populations [13]. Such validation requires appropriate statistical design and interpretation. Typical power calculations indicate whether a given sample size has any realistic chance of detecting bias. Furthermore, incorrect interpretation of formal statistical comparisons between two groups (e.g., marginalized versus privileged classes) has been a persistent problem for decades. When a null hypothesis significance test fails to reject the null hypothesis, the conclusion of “no difference”—or that the null hypothesis is true—is erroneous. In the context of assessing fairness of an AI model, this mistake is analogous to concluding a model is fair without sufficient evidence. Moreover, this mistake is exacerbated by studies that are underpowered to detect bias (let alone conclude fairness) due to small sample sizes, rare events, or small disadvantaged groups.

To address this issue, we embed the assessment of fairness within the framework of statistical equivalence testing [18]. Often, our interest is not whether two populations or processes are exactly equal or not, but rather whether the difference between two populations or processes is within some reasonable margin or not. We use statistical equivalence tests with the intention of showing that an AI model’s performance is equivalent within a margin when comparing a disadvantaged (or marginalized) group relative to a privileged group. An important aspect of this framework is to specify how much difference between model performance is allowable to be considered a fair model. The approach puts the onus on the user to consider what differences are meaningful for *practical significance* instead of *statistical significance* [19], and it will lead to more sound statistical conclusions to determine fairness.

The standard use case of statistical equivalence tests is in the design of clinical trials with the intention of showing two treatments have the same effect [20]; but equivalence testing has also been applied in other fields such as ecology [21], chemistry [22], communication [23] and psychology [19]. Our application of equivalence testing to assess fairness of machine learning models follows more recent developments that used statistical methodology for uncertainty quantification of association measures in machine learning fairness research [24,25,26,27,28], but these studies did not use equivalence testing. Complementary to two works in this literature that do consider equivalence testing [29,30], we provide extended demonstrations within an oncology setting; consider metrics other than relative risk; discuss how, for the same sample size, certain model performance metrics will have more or less power than others; and also consider practical implications and relevance in the face of impossibility theorems that imply certain levels of bias may remain even after attempts to generate fair models.

## 2. Materials and Methods

### 2.1. Canonical Setup

We focus on the simple setup of modeling a binary outcome with a binary classification. That is, consider an AI or machine learning model that classifies patients for a binary outcome, such as diseased versus not diseased, or the occurrence versus non-occurrence of an adverse event. The model’s outputs are interpreted as two dichotomous predicted classes, or risk groups, of high risk and low risk (e.g., based on a threshold of an estimated probability of the outcome).

The standard performance metrics follow naturally from the confusion matrix (i.e., a contingency table with the binary outcome as the columns and the binary model output as the rows), and we considered the following “success” metrics (where a larger value is good): sensitivity (a.k.a. recall), specificity, positive predictive value (PPV, a.k.a. precision), and negative predictive value (NPV) [31,32]. We also examine the complements of these values, referred to as “failure” metrics (where a larger value is bad): false negative rate (FNR = 1 − sensitivity), false positive rate (FPR = 1 − specificity), false discovery rate (FDR = 1 − PPV), and false omission rate (FOR = 1 − NPV). Here, we do not include accuracy (or its complement, error rate) because it is not an informative metric for rare events, which are the norm across biomedical applications.

For simplicity, we compare model performance between two groups: a disadvantaged group and a privileged group. We use the terms “disadvantaged” and “privileged” throughout but recognize the relevant interpretation is context-specific. As the contrast, we use the relative risk (i.e., the ratio of metrics), consistently reporting performance metrics of the disadvantaged group relative to the privileged group. A relative risk of one indicates identical performance in the two groups. For “success” metrics, a relative risk less than one indicates that the model performs more poorly for the disadvantaged group than for the privileged group. For “failure” metrics, the direction reverses, and a relative risk less than one indicates better performance for the disadvantaged group (see Table S1 for more interpretations).

### 2.2. Choosing Equivalence Margins

The idea of statistical equivalence testing is to determine whether the difference between two groups falls within a prespecified boundary, called the *equivalence margin*. The choice of the margin is a matter of an investigator’s judgment and should be based on what is considered clinically relevant.

In the context of fairness testing of an AI model, the equivalence margin corresponds to the limits of what could be considered fair. Put another way, true differences falling outside the equivalence margin would be considered evidence of biased performance. For the purposes of this demonstration, we use the “four-fifths rule” to guide the choice of equivalence margins for fairness. The four-fifths rule is a guideline from the US Equal Employment Opportunity Commission, where if one demographic group is selected less than four-fifths (or 80%) as often as the most selected group in an employment process, it *may* be the *starting point* of an *investigation* of disparate impact [33]. The application of this rule to assess fairness of machine learning models has been critiqued as woefully inadequate, not least because it is one standard of beginning a (manual, qualitative) investigation, and very far from how machine learning has reductively used it as a necessary and sufficient decision threshold [34]. Here, we use the four-fifths rule to allow comparability with other machine learning work. In the methods below, to have an analogous (or reciprocally symmetric) margin on both sides of the null when using 4/5 = 0.80 for the lower margin, we use 1/0.80 = 1.25 as the upper margin.

For any specific application, we emphasize that an equivalence margin should be a substantive ethical and clinical decision rather than based on an analogy with a rule of thumb from a separate domain. In the discussion, we expand on this point.

### 2.3. Testing for Equivalence

**Figure 1:**
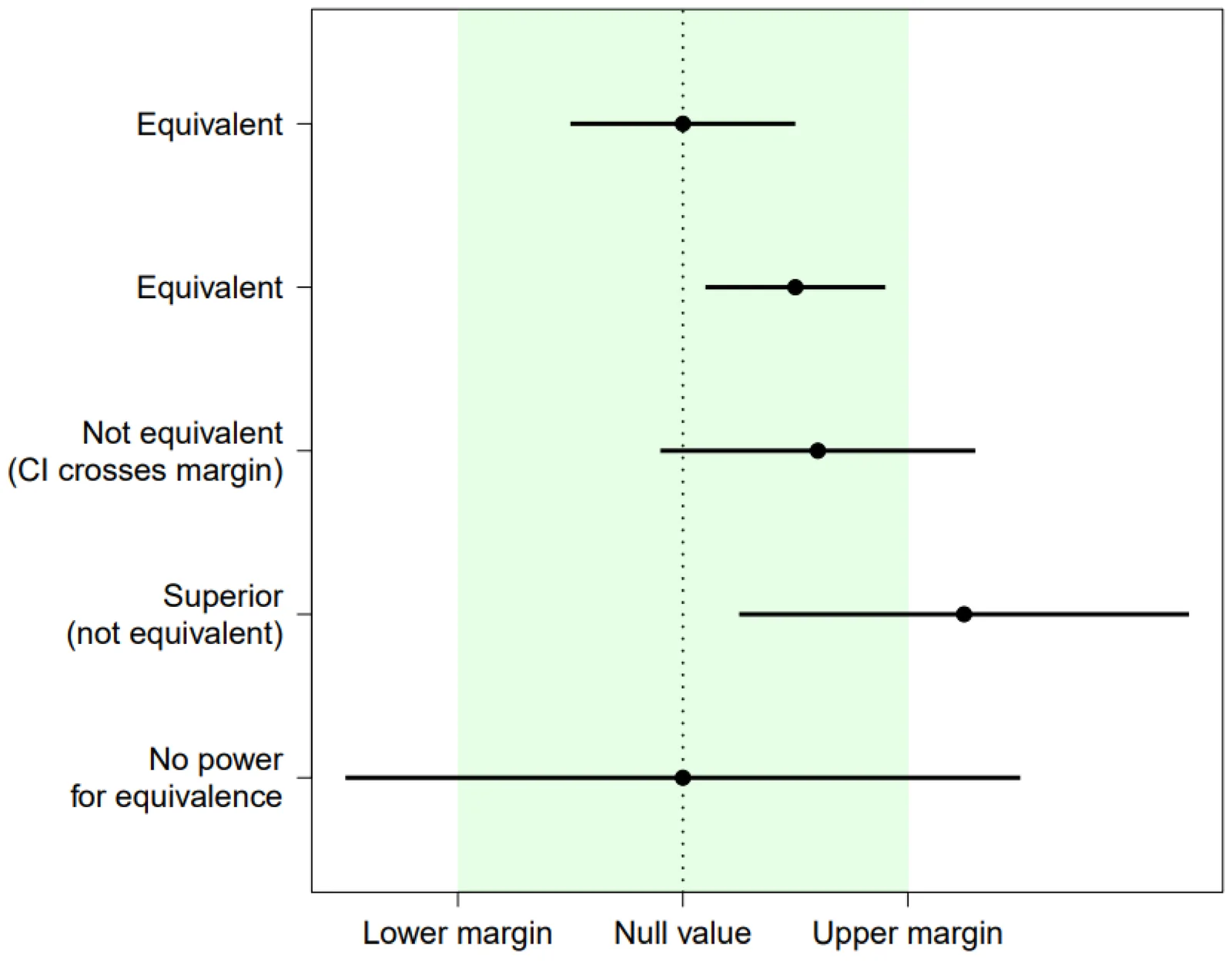
Diagram showing the conclusions from the equivalence tests for hypothetical estimates and 90% confidence intervals (CIs).

In order to conclude fairness or equivalence, all values outside the equivalence margin must be ruled out with a high degree of confidence that accounts for the inherent variability in the data. In practice, the decision reduces to whether a 90% confidence interval for the comparison of interest falls entirely within the equivalence margin [18]. Figure 1 shows examples of 90% confidence intervals and what the conclusion would be.

The conditions for equivalence can be framed in terms of null hypothesis statistical tests by using the “two one-sided tests” (a.k.a., TOST) approach [35]. As the name suggests, this method performs two one-sided tests for the lower and upper margins. Within our context for comparing relative risks, the two hypothesis tests can be formalized as follows:

H0_lower_: relative risk ≤ 0.80 versus H1_lower_: relative risk > 0.80

H0_upper_: relative risk ≥ 1.25 versus H1_upper_: relative risk < 1.25

Equivalence is concluded at the 5% significance level if H0_lower_ and H0_upper_ are both rejected at the 5% significance level. That is, rejecting both null hypotheses provides strong evidence for the respective alternative hypotheses, so the conclusion is that the true relative risk falls within the equivalence margin of 0.80 to 1.25. For a 5% significance level, the two one-sided tests procedure is equivalent to the 90% confidence interval procedure [36].

Using the two one-sided test method, power to detect equivalence can be calculated for a given sample size with a prespecified equivalence margin and an assumed effect size. Power calculations for equivalence trials are well-established and apply to a broad range of study designs, outcomes, and association measures [20]. Here, we apply these methods to the comparisons used in fairness assessments of AI models under our canonical setup. The power calculations are based on asymptotic approximations of the relative risk estimator, with the standard error formula derived for the log relative risk [37]. For the large sample approximations to hold, a conservative rule is to expect at least five counts in every relevant cell of the confusion matrices, and if these criteria are not met, more complex testing and confidence interval procedures are available (e.g., Koopman score interval, inverse hyperbolic sine interval, etc. [38]).

## 3. Results

### 3.1. Power Calculations Under “Utopian” Fairness

We first demonstrate the power calculations under an assumption of “utopian” fairness, which is an idealized scenario [39]. Here, utopian fairness assumes the disadvantaged group is not associated with prevalence (i.e., the proportion with the binary outcome of interest), sensitivity, and specificity (and, implicitly, also PPV and NPV). The rationale for considering this idealized and simplified scenario is that it allows us to show how variation in each of the specific parameters affects power while holding all other parameters constant. The specific parameters under consideration are the proportion in the disadvantaged group within the total population, the prevalence of the condition, the sensitivity, and the specificity (see Table 1 for specific values and default settings). Given prevalence, sensitivity, and specificity, the remaining metrics—PPV and NPV—are fixed and can be calculated via Bayes’ rule. For sample sizes ranging from 100 to 100,000, we calculate the power to detect equivalence within a relative risk margin of 0.80 to 1.25 under the assumption of utopian fairness.

**Figure 2:**
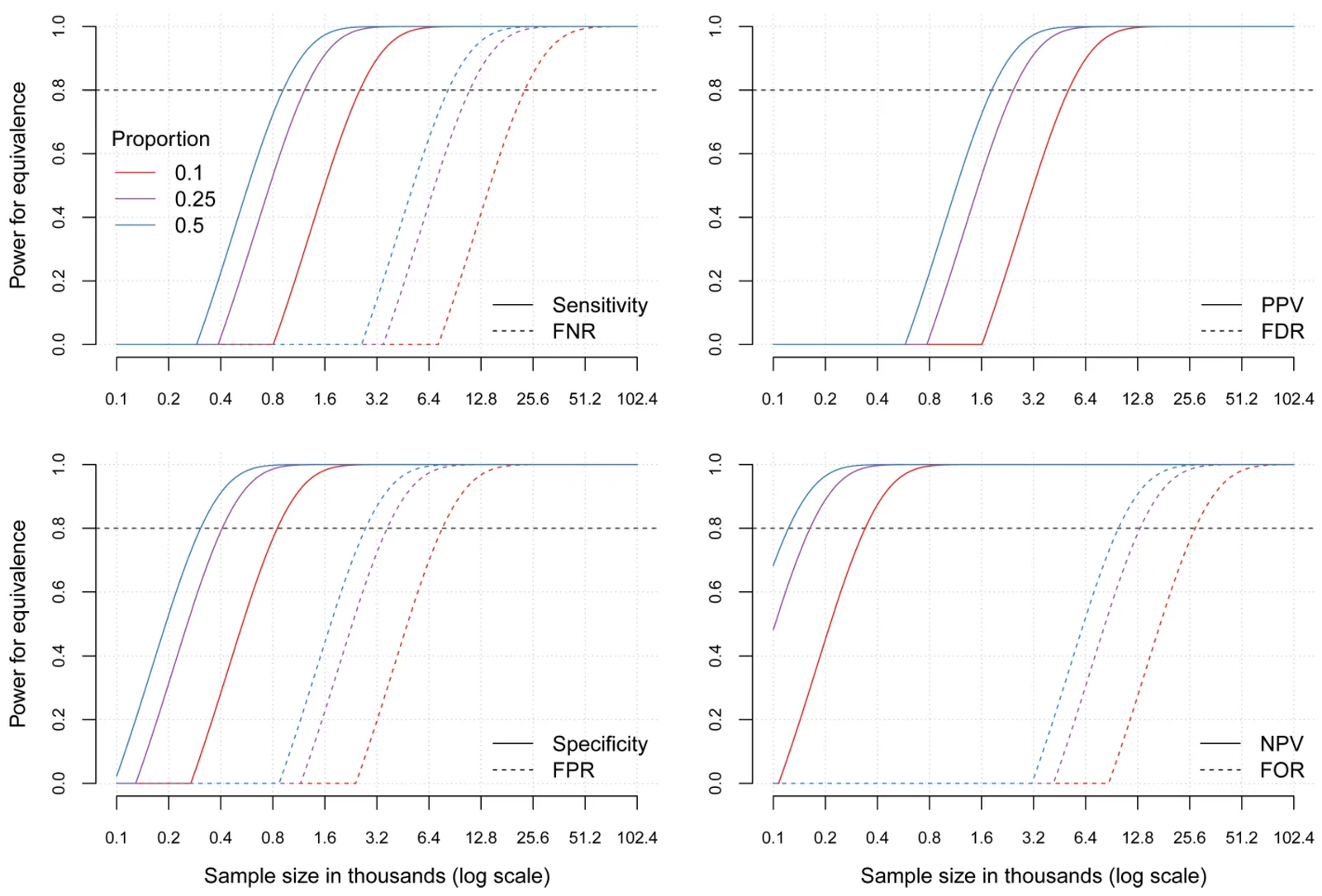
Power for equivalence as a function of sample size under various proportions in the disadvantaged group. Abbreviations: FNR, false negative rate; PPV, positive predictive value; FDR, false discovery rate; FPR, false positive rate; NPV, negative predictive value; FOR, false omission rate.

Figure 2 shows how power (as a function of sample size) for different performance metrics varies with the proportion in the disadvantaged group. For example, to obtain 80% power for equivalence for sensitivity, sample sizes of approximately 2600, 1200, and 900 are required when the proportion in the disadvantaged group is 0.10, 0.25, and 0.50, respectively. To obtain 80% power for specificity, the sample sizes can be smaller because under the default settings, the proportion with non-events is much larger (e.g., for a proportion of 0.25, a sample size of around 400 patients is required). For PPV, larger sample sizes are needed, with approximately 5100, 2500, and 1800 required to obtain 80% power when the proportion in the disadvantaged group is 0.10, 0.25, and 0.50, respectively. Like specificity, NPV requires smaller samples to show equivalence (e.g., for a proportion of 0.25, a sample size of fewer than 200 patients is required). Among the “success” metrics, the range of proportions from 0.1 to 0.5 impacts PPV the most, with the largest change in required sample size. The “failure” metrics require substantially larger sample sizes for the same power (except for FDR), which is discussed in more detail later (in Section 3.4). The primary takeaway from this figure, as is well known, is that a balanced comparison, that is, an equal split between privileged and disadvantaged groups, leads to the highest power (i.e., in a population of size N, both privileged and disadvantaged groups have sizes of approximately N/2).

**Figure 3:**
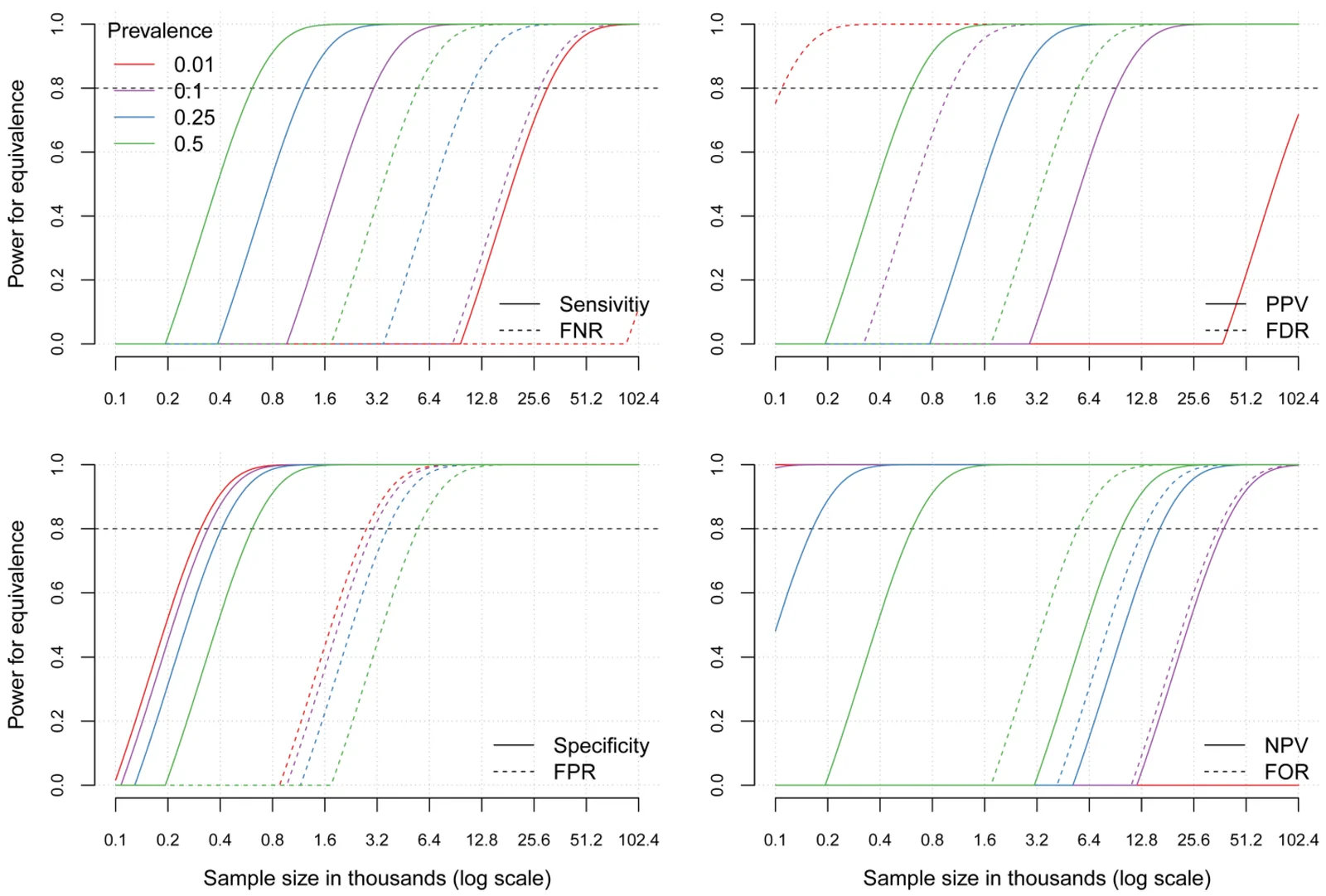
Power for equivalence as a function of sample size under various prevalence levels for the binary outcome of interest. Abbreviations: FNR, false negative rate; PPV, positive predictive value; FDR, false discovery rate; FPR, false positive rate; NPV, negative predictive value; FOR, false omission rate.

Figure 3 shows how power varies as the prevalence of the outcome varies. To obtain 80% power for comparing sensitivity, sample sizes of approximately 30,700, 3100, 1200, and 600 are required for prevalences of 0.01, 0.10, 0.25, and 0.50, respectively. Prevalence has less effect on power for specificity, with required sample sizes ranging from approximately 300 to 600 (for prevalences of 0.01 and 0.50, respectively). Models of rare outcomes therefore require substantially larger sample sizes to establish fairness with respect to sensitivity and PPV.

See Figures S1 and S2 for the effect of varying sensitivity and specificity, respectively.

### 3.2. Examples of Power Calculations Applied to Machine Learning Models in Oncology

**Figure 4:**
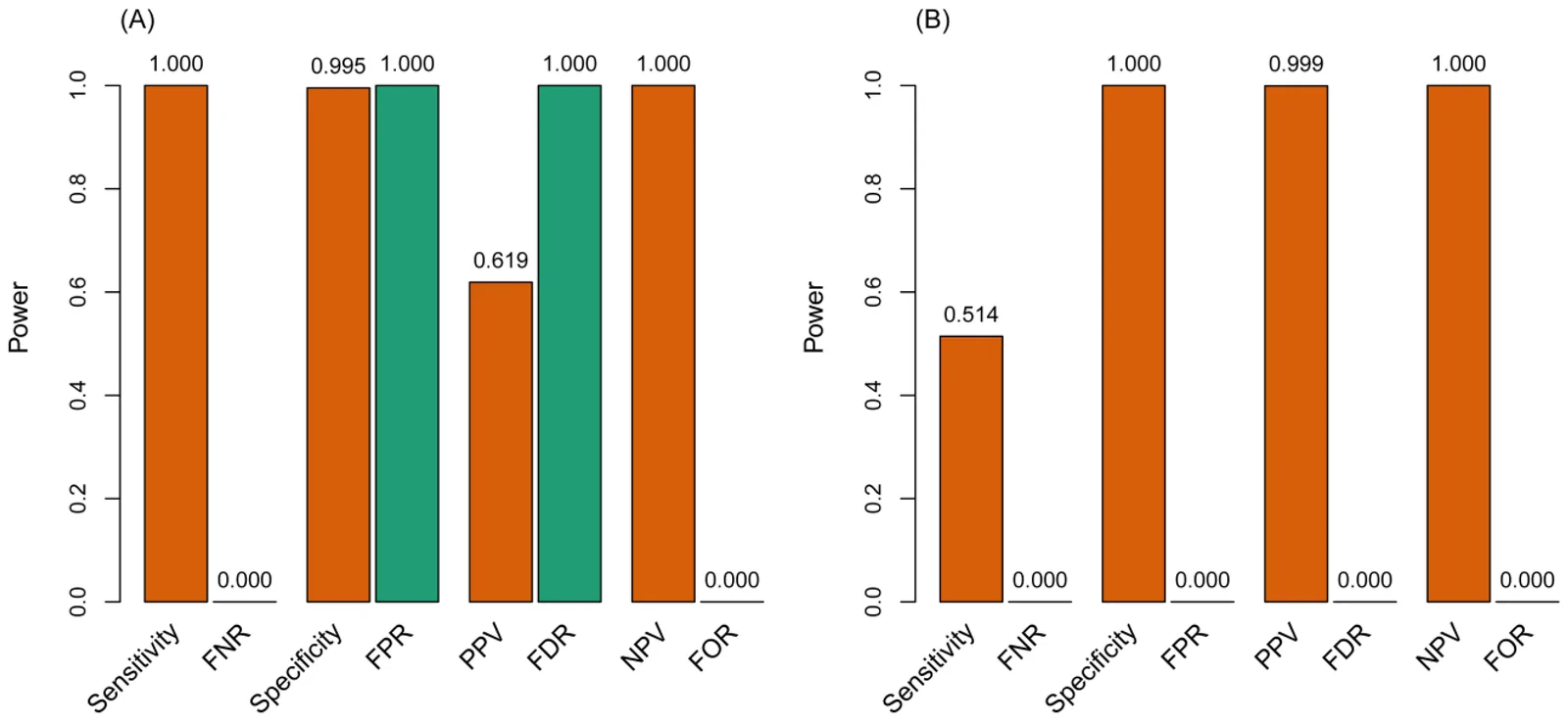
Power for equivalence under utopian fairness based on studies predicting recurrence among patients with colorectal cancer (**A**) and detecting *EGFR* mutation among patients with lung cancer (**B**). Orange indicates a “positive” metric, and green indicates the complementary “failure” metric.

We use a recent publication that examined strategies to mitigate racial and ethnic bias in risk prediction models for colorectal cancer recurrence [40]. We identified parameter values from this paper to demonstrate the power calculations but acknowledge the intent of that paper was more than a simple assessment of fairness between two groups. We used results from the prediction model that classified patients as either high- or low-risk for colorectal cancer recurrence that had a 3-year incidence rate of 0.112, and we compared 1981 non-White patients to 2249 (non-Hispanic) White patients (i.e., the proportion in the disadvantaged group is 0.468). Additionally, FNR was 0.073, and FPR was 0.699 (i.e., sensitivity was 0.927, specificity was 0.301, PPV was 0.143, and NPV was 0.970). Under these conditions, the sample size provides high power to conclude equivalence for sensitivity, specificity, FPR, FDR, and NPV, but limited power for PPV and essentially no power for FNR and FOR (Figure 4A).

As another example, we used summaries from a recent assessment of open-source AI models for detecting *EGFR* mutations from pathology slides among patients with lung cancer [41]. Among a specific cohort of N = 1465, model performance was compared between patients with European and Asian ancestry (proportion with Asian ancestry is 0.061). The prevalence of the *EGFR* mutation was 0.25, and model performance was reported as sensitivity of 0.84 and specificity of 0.99. With these settings, the sample size had high power to conclude equivalence for specificity, PPV, and NPV, but low (or no) power for the remaining metrics (Figure 4B).

Applied a priori, these calculations indicate which metrics and comparisons are likely to support a conclusion of fairness if the model does perform equally well for a given metric. Moreover, these calculations indicate which metrics cannot lead to conclusions of fairness and highlight the “no power” scenario illustrated in Figure 1 (i.e., even if the point estimate suggests the metrics are equal, the confidence interval is too wide to support any statistical conclusion about equivalence or fairness).

### 3.3. Practical Considerations with near 100% Power

Obtaining near 100% power—as both examples illustrate for certain metrics—is appealing insofar as equivalence will be concluded if it is true, but it may also indicate that a sample size is too large or an equivalence margin is too conservative. To allay the latter concerns, we emphasize that the results of a fairness assessment should focus on the observed point estimate and confidence interval instead of emphasizing the binary conclusion of statistically significant or not [42,43]. As a concrete example, if a relative risk from a fairness assessment had a 90% confidence interval of 0.98 to 1.04, it is statistically sound to descriptively note equivalence for the relative risk within the reciprocally symmetric margins 0.96 = 1/1.04 and 1.04 for the relative risk in addition to concluding fairness within the pre-specified margin of 0.80 to 1.25. Another consideration for situations with very high power is to consider subgroup analyses, which can ensure comparability between disadvantaged and privileged groups as well as highlight intersectional groups with poor performance [44,45,46].

Another practical approach is to hone the equivalence margin for different metrics. For the colorectal cancer example, fixing power for sensitivity at 90% allows the equivalence margin to narrow to 0.918 to 1.089, which would support a stronger conclusion of fairness. Fixing power for specificity at 90% allows an equivalence margin of 0.848 to 1.179. This approach demonstrates reverse engineering the margin when the sample size is already fixed (e.g., based on readily available data). However, we emphasize that the intended primary use is prospective in the study design phase, with the margin specified first and the sample size then chosen to provide adequate power for specific metrics.

### 3.4. Asymmetry of the Relative Risk

A notable feature of these power calculations is that, for a given set of parameter values, a “success” metric can have substantially different power than its complementary “failure” metric. This result is due to the asymmetry of the relative risk association measure. For example, under the default parameter settings, a sensitivity of 0.75 requires a sample size of approximately 1,200 to obtain 80% power to detect equivalence, whereas the FNR (i.e., 1 − sensitivity) of 0.25 requires a sample size of approximately 11,100 to obtain similar power (Table 2). The asymmetry can be considered with hypothetical point estimates; for example, if the sensitivity in the disadvantaged group was 0.75 and 0.90 in the privileged groups, then the relative risk of 0.83 is within the equivalence margin despite a fairly large difference in proportions; in contrast, the FNR in the disadvantaged group is 0.25 and 0.10 in the privileged group for a relative risk of 2.5, which is well outside the equivalence margin (see Appendix A for another heuristic numerical explanation via equivalence margins).

An alternative strategy is to use an association measure that is symmetric, for example, either the odds ratio or the risk difference. Our choice of the relative risk was based in part on the four-fifths rule, which corresponds naturally to a ratio of proportions. Relative risks are also often preferred across domains because they are easier to interpret (e.g., they correspond to statements like “the rate in condition *A* is twice the rate of condition *B*”). The same numerical margins of 0.80 to 1.25 could be applied to the odds ratio, but four-fifths on the odds scale is not equivalent to four-fifths on the risk scale (see Appendix A for heuristic numerical explanation). Applied to the odds ratio, these limits correspond to effects smaller than what is conventionally regarded as a small effect on the scale of standardized mean difference (i.e., a Cohen’s *d* of 0.2) [47,48]. In general, the odds ratio requires larger sample sizes than the relative risk for either the success or the failure metric (see Figure S3 for power calculations with odds ratios). In the prior example, using the odds ratio would require sample sizes of approximately 19,700 (for both sensitivity and FNR) to obtain 80% power for equivalence, which is far larger than the 11,100 needed for the relative risk of FNR (Table 2).

### 3.5. Impossibility of Fairness and Equivalence Power Under Small Bias

An important theoretical result in fairness assessment is the impossibility of simultaneously satisfying certain fairness criteria [39,49,50]. Namely, the impossibility result states that if a classification model meets properties of separation (i.e., exactly fair with respect to sensitivity and specificity) and sufficiency (i.e., exactly fair with respect to PPV and NPV), then either utopian fairness is true, or there is a degeneracy in the probability model (i.e., probabilities of 0). The assumption of utopian fairness is an idealization that is unrealistic in practice, so relaxing it is important to consider.

For example, if the prevalence of the outcome event varies by the sensitive trait, then both separation and sufficiency cannot hold in general. Table 3 presents values of PPV and NPV when sensitivity and specificity are equal between disadvantaged and privileged groups, but prevalence differs between the disadvantaged and privileged groups (under default values reported in Table 1). A higher prevalence in the privileged group leads to worse relative performance for the disadvantaged group with respect to PPV. The true relative risk for PPV is still within the equivalence margins, but power decreases sharply as the relative risk approaches the equivalence boundaries. In contrast, the impact on NPV is only modest, leading to a relatively small decrease in power.

For an empirical demonstration of the impossibility theorem, we return to the model predicting colorectal cancer recurrence [40]. For the outcome of recurrence, there was evidence of a difference in rates of recurrence by race. Specifically, the prevalence was 0.131 in the non-White group and 0.095 in the (non-Hispanic) White group. When sensitivity and specificity are assumed to be the same across racial groups, the differential prevalence leads to better performance for the disadvantaged group with respect to PPV (i.e., 0.167 for non-White versus 0.122 for White) with a relative risk of 1.36, and worse performance with respect to FOR (i.e., 0.0353 for non-White versus 0.0248 for White) with a relative risk of 1.42; both of these ratios are already outside the equivalence margin. In contrast, the changes in relative risk for FDR and NPV are only modest deviations from the fair (or null) value of one, and the power is unaffected.

The practical implications of the impossibility results are that exact fairness for all metrics is generally not obtainable in practice. The machine learning literature has addressed this issue by imposing fairness constraints within specified limits [51,52]. Statistical equivalence testing is a natural analog of this approach, relaxing the definition of fairness to allow performance to differ by only so much. The distinction between the approaches is that the machine learning strategies for mitigating bias build the fairness constraints into the model, whereas equivalence testing is a formal test used to validate fairness empirically.

## 4. Discussion

We have described the notion of fairness in the context of statistical equivalence testing. A key takeaway is that standard (single test) null hypothesis tests should not be used to conclude that an AI model is fair, because they cannot provide strong evidence for the null hypothesis. Rather, equivalence testing provides a framework for when to conclude equivalent model performance based on prespecified and clinically relevant effects. We used the four-fifths rule for our boundary conditions, but any specific application should adopt more or less stringent margins based on substantive input. We used oncology as a motivating example with empirical illustrations, but this approach is disease-agnostic and can be readily applied to fairness assessments in other medical contexts.

Another takeaway is that adequate power for all performance metrics is often not attainable. Thus, a well-designed fairness study should prespecify the primary metrics of interest with substantive equivalence margins and then target a cohort with a sample size sufficient to provide reasonable power to show fairness. Which metrics should be prespecified depends on the clinical consequence of the errors the model makes: sensitivity and FNR are the primary concern when a missed case carries the greater harm, whereas specificity, FPR, and PPV are the primary concern when the burden of unnecessary follow-up dominates, and FOR captures who is left out of a potentially assistive intervention [53]. Such an intentional design forces investigators to provide a priori criteria for success of a fairness assessment akin to the rigor required for designing clinical trials. In contrast, a less intentional but more pragmatic fairness assessment might use a convenience sample of readily available data, in which case, these power calculations can inform which metrics have a reasonable chance of concluding equivalence versus those that do not. In both cases, the equivalence framework provides clear rules that avoid concluding an AI tool is fair for metrics that are estimated with substantial uncertainty.

A limitation of this work is our focus on a canonical setup with simple comparisons of standard confusion matrix metrics based on binary outcomes, binary classification, and comparison of two groups. We chose this simplified framework because it is also common, and for the sake of interpretability and replicating how such an AI tool might ultimately be implemented into practice. However, we emphasize the importance of other modeling scenarios and metrics for calibration and discriminative ability. One challenge for defining equivalence for such metrics is having an interpretable equivalence margin. For example, comparing area under the receiver operating characteristic curves [54] (AUCs) is often used to compare discriminative abilities of a classification model; and indeed, the example of detecting *EGFR* mutation among lung cancer patients showed differential performance with respect to AUC. However, it is not clear which equivalence margins for differences in AUC would be interpretable or useful, and other work has detailed further nuances that complicate the use of AUC in fairness assessments [55]. Other aspects that remain to be addressed include multiclass or multilabel outcomes with top-*k* labels and comparisons involving more than one disadvantaged (or sensitive) group. Developing the latter scenario could address the potential issue of intersectional subgroups, which has important historical precedent in discrimination law (i.e., considering the impact of the interaction of race and gender on hiring practices) [56,57]. Extending the application of equivalence testing for fairness to these modeling scenarios is an area for future research.

Given the limitations of applying the four-fifths rule outside its original context, substantive recommendations are needed for either standard margins or for how to determine margins in a specific use case. We acknowledge that clinicians may not know how to reason about risk ratio effect sizes or equivalence margins around model fairness; this might be a topic for future qualitative and ethical work, but in the meantime, we can suggest reasoning using concrete numbers. For example, if an AI model detects 95 out of 100 cancers in one group (sensitivity = 0.95), but only 76 out of 100 cancers in another group (sensitivity = 0.76), then the risk ratio is 0.76/0.95 = 0.8. But such a model, that is worse by 19 out of 100 cases, would likely be clinically and ethically unacceptable. If an acceptable margin would be no more than 5 fewer (i.e., no fewer than 90 out of 100 cancers diagnosed in the other group), then it would suggest a 0.90/0.95 = 0.947 effect size for an equivalence margin, which we could round to a 0.95 relative risk threshold to get a “nineteen-twentieths” effect size to use as an equivalence margin. Other more interpretable statements for clinicians might use metrics like the number needed to treat (a.k.a., NNT), which maps directly onto risk differences (i.e., differences in proportions) [5]. Prior descriptions of equivalence testing provide prescriptive approaches to defining subjective justification for equivalence margins, including using a trivially small effect size (e.g., Cohen’s *d* of 0.1), basing the margin on related studies in the literature, or basing the margin on previously assumed effect sizes (e.g., 33% of the effect used to power a superiority trial) [19]. However, this work also warns against “thoughtless application of ‘cookbook’ prescriptions” to define equivalency [58], again emphasizing that there is no single *procedure*, either, that can always be used: choices should be substantive and case-specific. In terms of our demonstrations, while we show things with a four-fifths margin, tighter margins would result in an increased needed sample size the same way that increased power requires increased sample size, but the asymmetries of risk ratios and the greater needed sample size for odds ratios remain the same.

Given the inherent variability in statistical precision across metrics, it makes sense to allow different equivalence margins for different metrics, which we demonstrated in the machine learning example for predicting colorectal cancer recurrence. One challenge for setting such margins is the explicit acknowledgment that perfect (or idealized) fairness may not be obtainable, especially in the context of the impossibility theorems for fairness. Such bias—even if small—may compound injustice, especially if applied at a large scale [59], making an argument for strict equivalence margins. An important acknowledgment is the potential for performance-fairness trade-offs, with more fair models leading to worse-performing models, and the challenge of balancing this trade-off is an ethical consideration and cannot necessarily be statistically informed [60].

Finally, we emphasize that assessing fairness in performance metrics of AI models does not address all sources of inequity. A complete assessment of fairness in AI should also address who has access to, and who benefits from, the implementation of these models; the systems within which these models are embedded; the kinds of actions taken on the basis of the models, that potentially depart in their effects from the performance of the models in nonrandom ways; and what systems of actionability and accountability might follow from a quantitative assessment of fairness [61,62,63,64,65,66,67,68,69].

## 5. Conclusions

This work addresses a practical gap in fairness assessments: investigators often report subgroup performance metrics without determining whether their sample size can distinguish meaningful bias from statistical noise or whether the evidence is strong enough to conclude that performance is comparable across subgroups. We propose an equivalence testing framework for fairness assessment that combines prespecified fairness margins, confidence intervals for comparisons, and power (or sample-size) calculations for various performance metrics.

## Figures and Tables

**Table 1:** Parameter settings for power calculations under utopian fairness. Bold values correspond to default values as one parameter is varied.

Parameter	Values
Proportion in disadvantaged group	0.10, **0.25**, 0.50
Prevalence of outcome in the population	0.01, 0.10, **0.25**, 0.50
Sensitivity	0.50, **0.75**, 0.90
Specificity	0.50, **0.75**, 0.90
Equivalence margin	**0.80–1.25**

**Table 2:** Comparison of asymmetric relative risk versus symmetric odds ratios for sample sizes required for 80% power for equivalence of sensitivity and FNR under default scenarios in Table 1.

Effect Measure	Performance Measure	Sample Size for 80% Power
Relative risk	Sensitivity	1230
Relative risk	FNR = 1 − sensitivity	11,067
Odds ratio	Sensitivity and FNR	19,672

**Table 3:** Implied values of PPV and NPV in disadvantaged and privileged groups, assuming sensitivity and specificity (i.e., 0.75) are the same in both groups, but prevalence varies.

Prevalence		PPV				NPV			
Disadv.	Priv.	Disadv.	Priv.	RR	Power for N = 2450	Disadv.	Priv.	RR	Power for N = 164
0.25	0.25	0.500	0.500	1.000	0.80	0.900	0.900	1.000	0.80
-	0.26	-	0.513	0.974	0.78	-	0.895	1.005	0.79
-	0.27	-	0.526	0.951	0.72	-	0.890	1.011	0.78
-	0.28	-	0.538	0.929	0.63	-	0.885	1.017	0.76
-	0.29	-	0.551	0.908	0.52	-	0.880	1.023	0.75
-	0.30	-	0.562	0.889	0.41	-	0.875	1.029	0.73

## Data Availability

The original contributions presented in this study are included in the article. Further inquiries can be directed to the corresponding author.

## Supplementary Materials

The following supporting information can be downloaded at: https://www.mdpi.com/article/10.3390/cancers18213514/s1, Figure S1. Power for equivalence as a function of sample size under various sensitivity values; Figure S2. Power for equivalence as a function of sample size under various specificity values; Figure S3. Power for equivalence as a function of sample size under various proportions in the disadvantaged group for both relative risks and odds ratios; Table S1. Interpretation of relative risks.

## Abbreviations

The following abbreviations are used in this manuscript:

AI	Artificial intelligence
AUC	Area under the receiver operating characteristic curve
FDR	False discovery rate
FNR	False negative rate
FPR	False positive rate
FOR	False omission rate
NPV	Negative predictive value
PPV	Positive predictive value

## Appendix A

### Numerical Heuristic Demonstrating the Asymmetry of Relative Risks in Contrast to the Symmetry of Odds Ratios

If we assume that the true sensitivity for the privileged group is known to be 0.75, then applying the 0.80 to 1.25 equivalence margin for the relative risk is the same as showing that the sensitivity for the disadvantaged group falls between 0.60 and 0.94. By the same argument, the FNR for the disadvantaged group must fall between 0.20 and 0.31 to show equivalence. In both cases, we are effectively estimating the same proportion, but a much smaller sample size is required to show a proportion falls within the 34-percentage point equivalence margin for sensitivity than the 11-percentage point equivalence margin for FNR.

A similar argument shows how different the margin is when applying the equivalence margin to the odds ratio instead of the relative risk. If the true sensitivity for the privileged group is 0.75 (or odds of 3), the equivalence margin of 0.80 to 1.25 on the odds scale corresponds to a sensitivity for the disadvantaged group ranging from 0.71 to 0.79 (or an FNR ranging from 0.21 to 0.29). This range is even smaller than the range for the FNR, only 8 percentage points, which corresponds to requiring an even larger sample size for the equivalence test applied to the odds ratios (Figure S3).
