# Supplementary material for: Addressing Power of Equivalence Tests for Statistical Fairness Assessments of Machine Learning Models in Healthcare
Source: Cancers (Basel). 2026 Oct 31;18(21):3514. doi: 10.3390/cancers18213514 (PMC13761817; doi:10.3390/cancers18213514)
Supplement: Supplementary file 1 [file cancers-18-03514-s001.zip › cancers-4598970-supplementary.pdf]

Figure S1. Power for equivalence as a function of sample size under various sensitivity values.

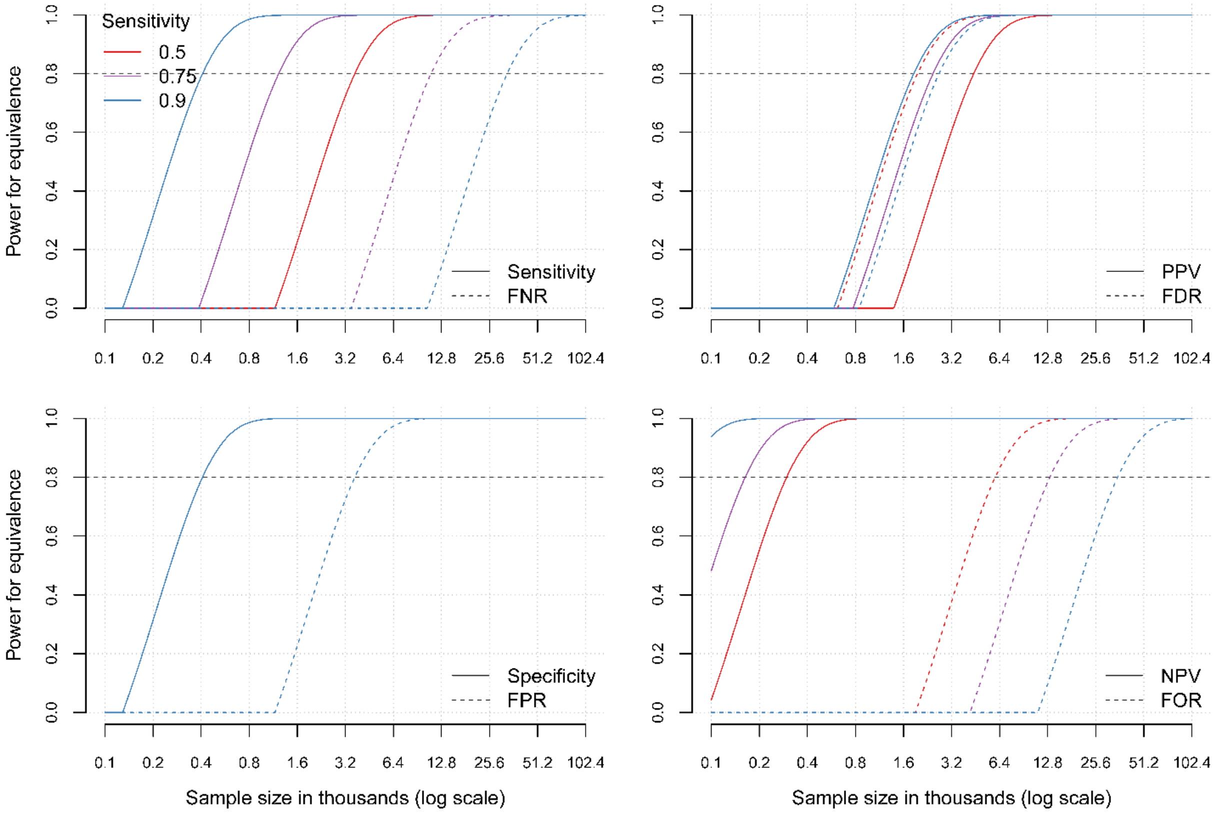

Figure S2. Power for equivalence as a function of sample size under various specificity values.

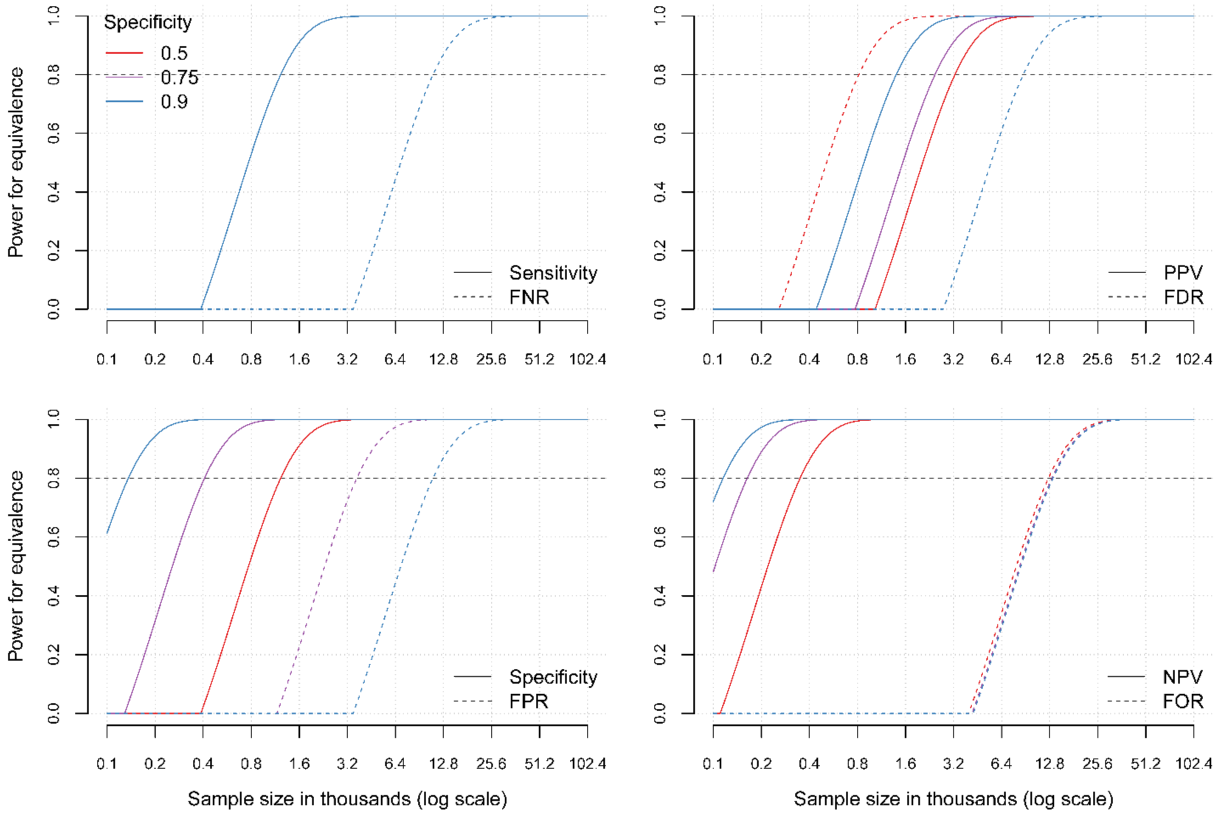

Figure S3. Power for equivalence as a function of sample size under various proportions in the disadvantaged group for both relative risks and odds ratios.

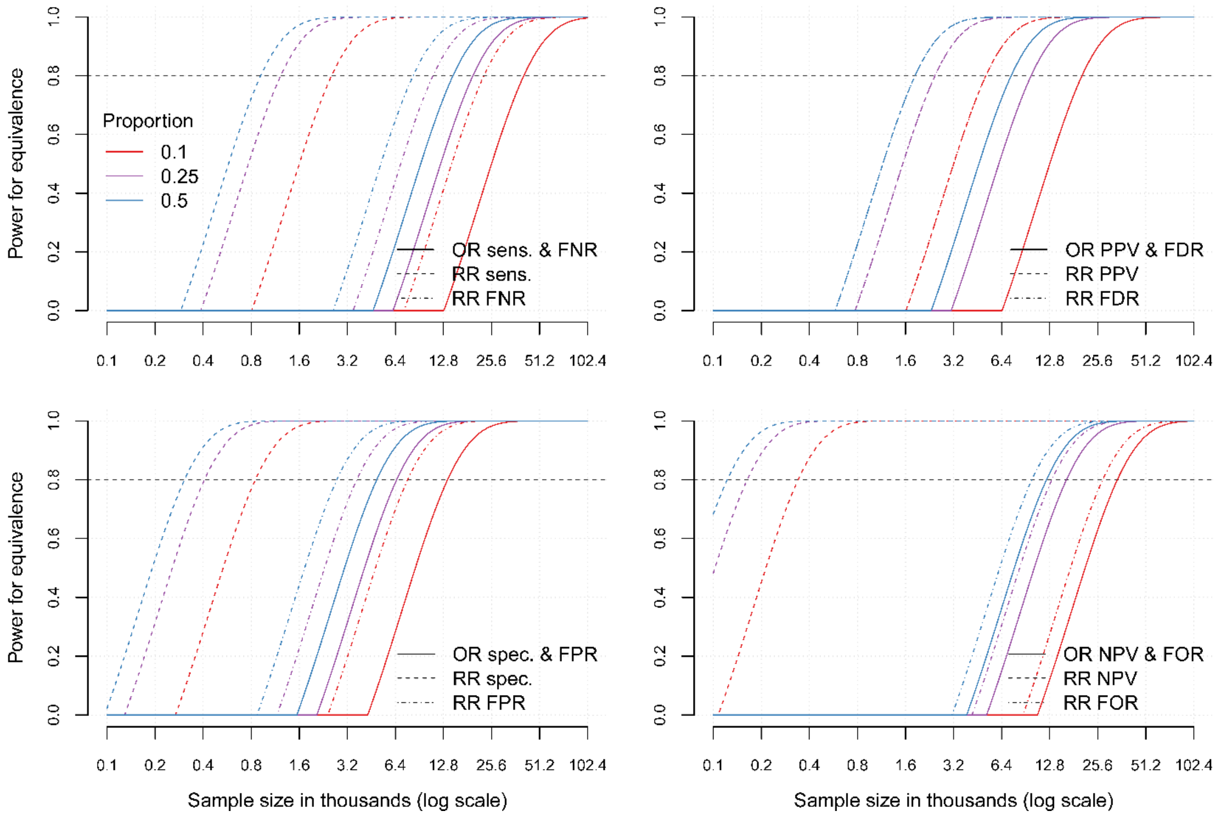

Table S1. Interpretation of relative risk. This table describes how to interpret direction of effect for relative risk across different combinations of type of metric, which group is in the numerator versus in the denominator, and the direction of the ratio relative to 1. The shaded rows are what we use as a canonical setup.

Type	Metrics	Numerator	Denominator	Relative risk	Favors
Success	Accuracy, Sensitivity, Specificity, PPV, NPV	Privileged	Disadvantaged	Greater than 1	Privileged
		Privileged	Disadvantaged	Less than 1	Disadvantaged
		Disadvantaged	Privileged	Greater than 1	Disadvantaged
		Disadvantaged	Privileged	Less than 1	Privileged
Failure	Error Rate, FNR, FPR, FDR, FOR	Privileged	Disadvantaged	Greater than 1	Disadvantaged
		Privileged	Disadvantaged	Less than 1	Privileged
		Disadvantaged	Privileged	Greater than 1	Privileged
		Disadvantaged	Privileged	Less than 1	Disadvantaged
